# Supplementary material for: Heterogeneity-stabilized homogeneous states in driven media
Source: Nat Commun. 2021 Jul 23;12:4486. doi: 10.1038/s41467-021-24459-0 (PMC8302733; doi:10.1038/s41467-021-24459-0)
Supplement: Supplementary file 1 — Supplementary Information [file 41467_2021_24459_MOESM1_ESM.pdf]

# SUPPLEMENTARY INFORMATION

*Heterogeneity-stabilized homogeneous states in driven media*

Zachary G. Nicolaou, Daniel J. Case, Ernest B. van der Wee, Michelle M. Driscoll, and Adilson E. Motter

## SUPPLEMENTARY DISCUSSION 1

### Band gaps in a pendulum array with heterogeneous masses

Here, we consider an alternative pendulum model in which the masses of the pendula vary rather than the lengths. This case is of easy experimental implementation and illustrates the generality of band gap opening by periodic heterogeneities (see Methods). The equations of motion for the driven array are given by

$$M_i L\ddot{\theta}_i = -\eta L\dot{\theta}_i - M_i[g - a_d\omega_d^2\cos(\omega_d t)]\sin(\theta_i) + \kappa L[\sin(\theta_{i+1} - \theta_i) + \sin(\theta_{i-1} - \theta_i)], \quad (1)$$

where $1 \leq i \leq N$. In the absence of driving ($a_d = 0$), linearization around $\theta_i = 0\ \forall i$ leads to an expression in the form of (9):

$$M_i\ddot{\theta}_i + \eta\dot{\theta}_i + \sum_j \left[(M_i g/L + 2\kappa)\delta_j^i - \kappa(\delta_j^{i+1} + \delta_j^{i-1})\right]\theta_j = 0, \quad (2)$$

where the heterogeneity is determined by the differing $M_i$. Supplementary Fig. 1 shows how wave modes split into distinct branches for a specific periodic heterogeneity with a three-particle unit cell,

$$M_i = \begin{cases} 1 - \lambda & i \bmod 3 = 0, \\ 1 & i \bmod 3 = 1, \\ 1 + \lambda & i \bmod 3 = 2, \end{cases} \quad (3)$$

with $L = 1$, $g = 1$, and $\kappa = 1$. Similar to the pendulum array discussed in the main text, when this system is parametrically driven with driving frequencies around twice the frequencies in a band gap, there are no resonant wave modes that can be easily excited, and thus heterogeneity-stabilized homogeneous states emerge.

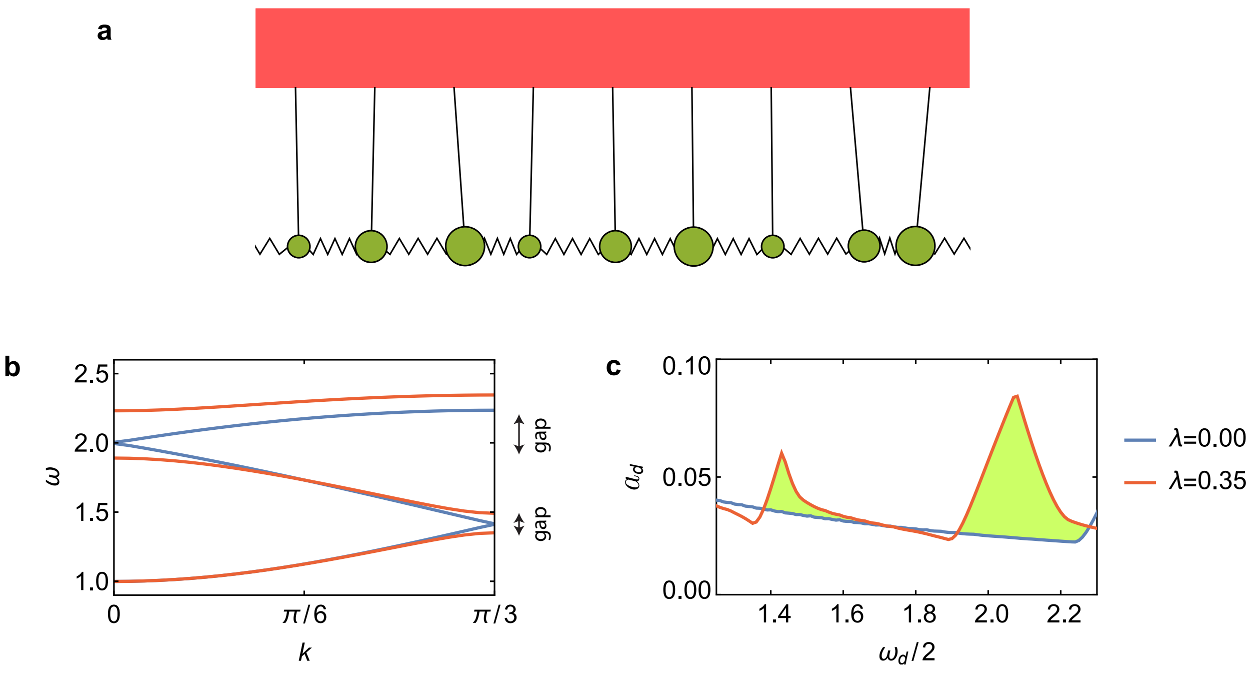


**Supplementary Fig. 1. Band-gap opening and HSHS for the pendulum array with heterogeneous masses.** **a**, Schematic of the pendulum array with a three-particle unit cell described by Supplementary Eqs. (1)-(3). **b**, Frequency $\omega$ vs. wavenumber $k$ for $\lambda = 0$ (blue lines) and $\lambda = 0.35$ (red lines) in the $N \to \infty$ limit for the system shown in **a**. Two gaps open between the three branches in the heterogeneous case. **c**, Instability boundaries for the systems shown in **b**, with green shading showing areas exhibiting HSHS associated with the respective band gaps in **b**.

## SUPPLEMENTARY DISCUSSION 2

### Finite-size perturbations and gap solitons in driven pendulum arrays

In the main text, we established that the homogeneous states are stabilized in the band gaps when heterogeneity is introduced, in the sense that infinitesimal perturbations around them decay. However, other stable states may also emerge when the system is driven. If multiple stable states coexist, sufficiently large perturbations may induce transitions between states even when the initial state is stable against infinitesimal perturbations. Here, we consider heterogeneity-stabilized homogeneous states in the presence of finite-size perturbations. For concreteness, we focus on the driven pendulum array with periodic heterogeneity considered in the main text, which is defined by alternating pendulum length $L_i = \overline{L} + (-1)^i \Delta$.

We find that alternative stable states do, in fact, emerge in the periodic pendulum array in parameter regions that exhibit heterogeneity-stabilized homogeneous states, as shown in Supplementary Fig. 2. This follows because the instability of any particular wave mode becomes subcritical when the driving frequency is smaller than the resonant frequency of that mode (left panel in Supplementary Fig. 2a), which should be contrasted with the supercritical form of the instabilities for larger driving frequencies (right panel in Supplementary Fig. 2a). The difference between the driving frequency and the resonant frequency of a particular mode is called the detuning parameter for that mode, so modes exhibit subcritical instabilities for negative detuning parameters. In the subcritical cases, an unstable swinging state vanishes at a *secondary* instability boundary of a new stable periodic swinging state that emerges for increasing driving amplitude. Since the detuning

parameter for the mode is negative in this case, there exists a different, lower frequency mode that is resonant with the driving frequency. Thus, the secondary instability boundary for the swinging states may lie above or below the (primary) instability boundary for the uniform state, which consists of the envelope of the instability boundaries over all modes.

The central panel in Supplementary Fig. 2a shows the primary instability boundaries for the pendulum array in the $N \to \infty$ limit with $\Delta = 0$ and $\Delta = 0.35$ (solid lines). The dashed lines show the instability boundary for the $k = \pi/2$ mode that is split by the bad gap, the dotted lines show the secondary instability boundaries for this mode in the subcritical cases. For the homogeneous array (orange lines), the secondary instability boundary for this mode lies above the

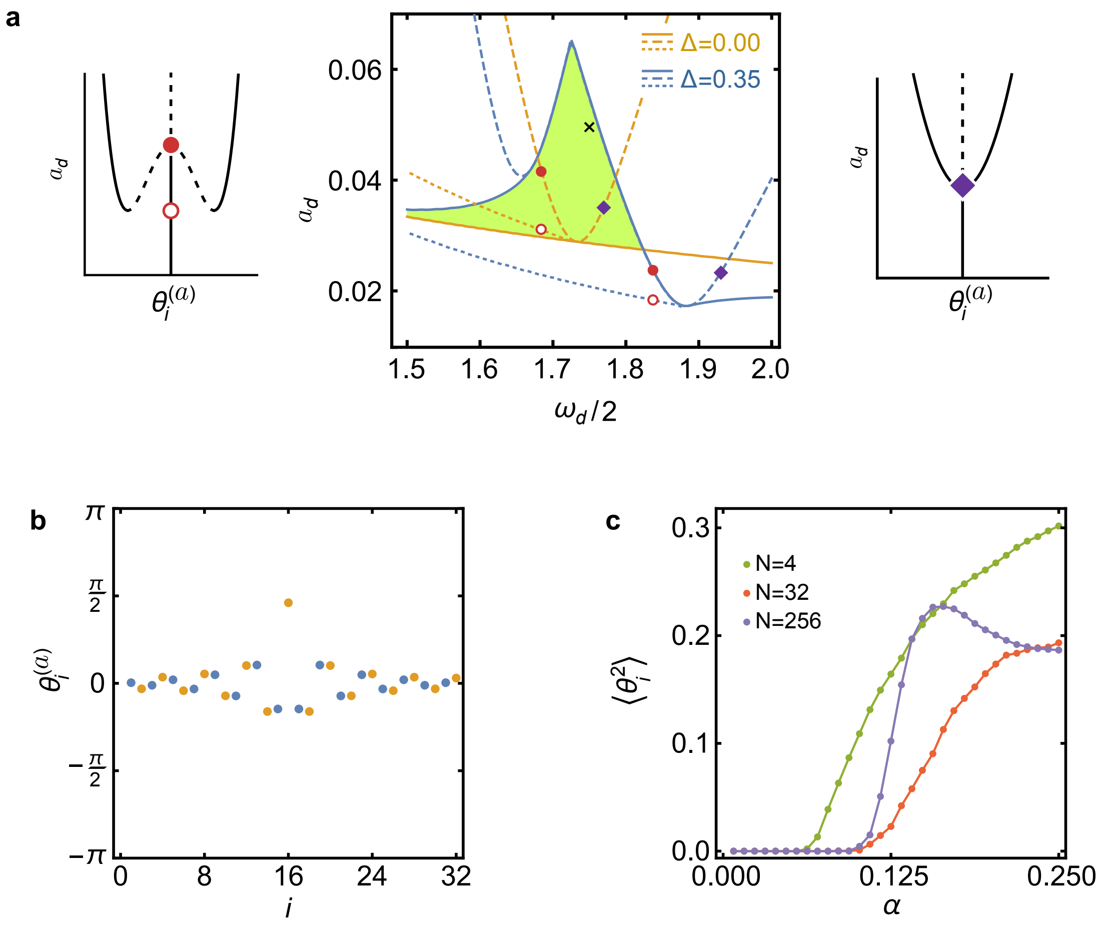


**Supplementary Fig. 2. Multistability and finite-size perturbations in pendulum array model.** **a**, Primary instability boundary for the homogeneous array (solid orange line) and heterogeneous array with $\Delta = 0.35$ (solid blue line) in the $N \to \infty$ limit. The boundaries for the $k = \pi/2$ instability mode (dashed lines) and the corresponding secondary instability boundary (dotted lines) are also shown, along with the region of HSHS (green area). Outsets: driving amplitude vs. swinging amplitudes for negative detuning parameters (subcritical bifurcation, left panel) and positive detuning parameters (supercritical bifurcation, right panel). Solid lines show stable states, dashed lines show unstable states, and symbols (also marked in the central panel) indicate the instability boundary (filled circles and diamonds) and the secondary instability boundary (open circles) for a particular mode. **b**, Swinging amplitude vs. pendulum index for a localized gap soliton state in a heterogeneous array determined by the parameters marked by $\times$ in **a**, with short pendula shown as orange dots and long pendula shown as blue dots. **c**, Average (over time and perturbation realization) of the squared phases of final stable state $\langle\theta_i^2\rangle$ vs. perturbation size $\alpha$ for heterogeneous arrays of various size with the other parameters corresponding to $\times$ in **a**.

primary instability boundary, so the system is not susceptible to finite-size instabilities below the primary instability boundary. For the heterogeneous array in this figure (blue lines), on the other hand, the secondary instability boundary for this mode lies below the primary instability boundary, and finite-size perturbations can therefore excite the array to the periodic swinging state below the primary instability boundary. In the outsets, the $\theta_i^{(a)}$ describe the swinging state amplitudes, defined by the value of the phases at the time points where $\sum_i \theta_i^2$ is maximized.

Interestingly, we find that the periodic swinging states are not the only alternative stable states for the heterogeneous array. Supplementary Fig. 2b shows the swinging state amplitudes for a particularly interesting localized stable state that we observe for random initial conditions in an array of 32 pendula for the parameter values marked by the $\times$ ($\omega_d = 3.5$ and $a_d = 0.05$) and heterogeneity in Supplementary Fig. 2a. An animation of this localized state is also available as part of Supplementary Movie 1. Since these localized states can coexist in a variety of spatial configurations, the heterogeneous pendulum array exhibits a high degree of multistability. Similar gap soliton solutions have been observed around band gaps in other media, emerging through a snaking bifurcation in pattern-formation models [S1]. While beyond the scope of this work, we expect that such gap solitons will also exist in Faraday instability systems with periodic substrates within regions of HSHS. We argue that this is expected because the bifurcations of instability modes have been shown to become subcritical for negative detuning parameters in Faraday wave experiments with homogeneous substrates [S2], in direct analogy with the orange lines in Supplementary Fig. 2a.

Finite-size perturbations can induce a transition between the homogeneous state and the stable swinging states below the instability boundary. To quantify the stability against finite-size perturbations, we simulate the system with a random initial perturbation given by an initial $\theta_i$ uniformly distributed in $[-\alpha\pi, \alpha\pi]$ and an initial $\dot{\theta}_i = 0$. Here, $\alpha$ quantifies the size of the perturbation. The system is evolved until it approaches a stable state. Supplementary Fig. 2c shows the time-averaged value of the phases (after the decay of the initial transient) averaged over 1024 random perturbation realizations for arrays of various sizes for the parameter values shown by the $\times$ in Supplementary Fig. 2a. The stability transition sharpens as the number of pendula increases and, for large $N$, the homogeneous state is stable against almost all simulated finite-amplitude perturbations for $\alpha < 0.1$. Thus, finite-size perturbations can destabilize heterogeneity-stabilized homogeneous states and lead to nontrivial dynamical states, but doing so requires large perturbations.

[S1] Ponedel, B. C. & Knobloch, E. Gap solitons and forced snaking. *Phys. Rev. E* **98**, 062215 (2018).
[S2] Cross, M. C. & Hohenberg, P. C. Pattern formation outside of equilibrium. *Rev. Mod. Phys.* **65**, 851 (1993).
