# Supplementary material for: Heterogeneity-stabilized homogeneous states in driven media
Source: Nat Commun. 2021 Jul 23;12:4486. doi: 10.1038/s41467-021-24459-0 (PMC8302733; doi:10.1038/s41467-021-24459-0)
Supplement: Supplementary file 2 — Description of Additional Supplementary Files [file 41467_2021_24459_MOESM2_ESM.docx]

Description of Additional Supplementary Files

Title: Supplementary Movie 1

Description: Heterogeneity-stabilized homogeneous states and gap solitons in the driven pendulum array. First segment: Pendulum dynamics for the arrays in Fig. 2a subject to identical driving defined by $\omega_{d} =3.5$ and $a_{d}=0.05$. The homogeneous array undergoes a symmetry-breaking instability while the periodic and random arrays exhibit heterogeneity-stabilized homogeneous states. Second segment: Localized gap soliton states exhibited by the heterogeneous arrays for the same driving when the pendulum angles are subject to a large random perturbation (uniformly distributed in the interval $[-0.4, 0.4]$). After the initial transient, the dynamics of the homogeneous array remain qualitatively unchanged upon this perturbation.

Title: Supplementary Movie 2

Description: Excerpt recordings from the Faraday instability experiments. The flat (top), sinusoidal (middle), and random (bottom) substrates are driven by identical vibrations at the three different frequencies and amplitudes described in Fig. 4c. For each driving condition, Faraday waves develop for the flat substrate but the fluid surface remains flat for the sinusoidal and random substrates, which demonstrates that heterogeneity can stabilize homogeneous states.
